# Supplementary figures and images for: Spatially selective cell treatment and collection for integrative drug testing using hydrodynamic flow focusing and shifting
Source: PLoS One. 2023 Jan 17;18(1):e0279102. doi: 10.1371/journal.pone.0279102 (PMC9844832; doi:10.1371/journal.pone.0279102)

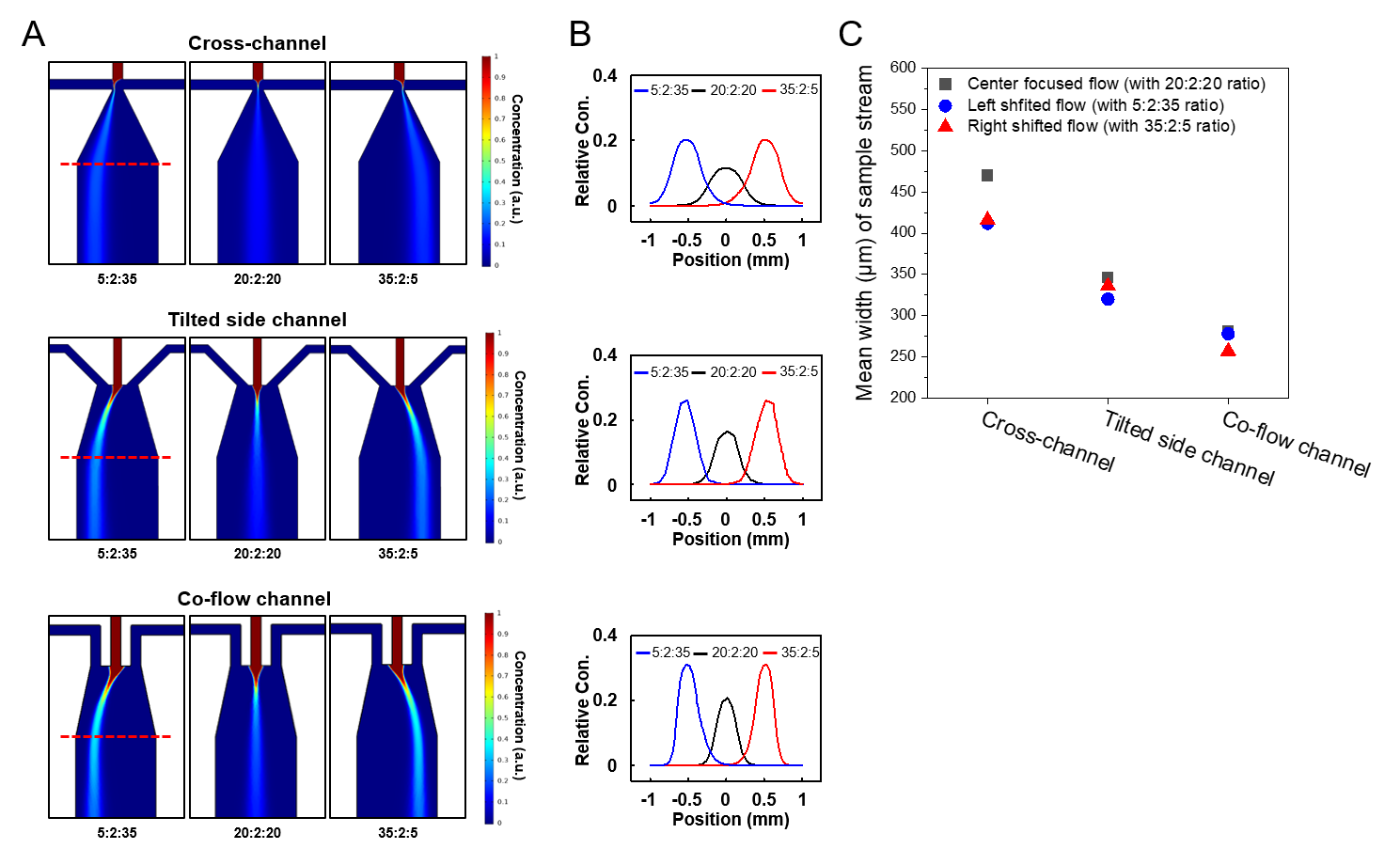

Supplement: S1 Fig — (TIF) [file pone.0279102.s001.tif]

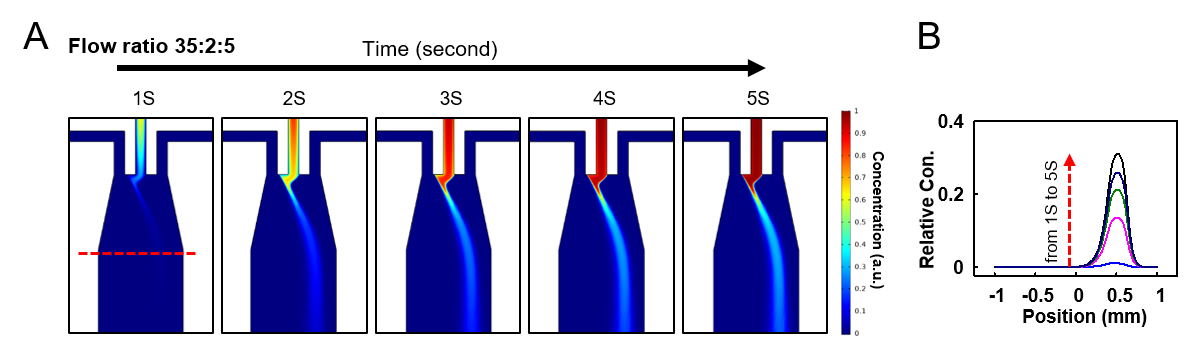

Supplement: S2 Fig — (TIF) [file pone.0279102.s002.tif]

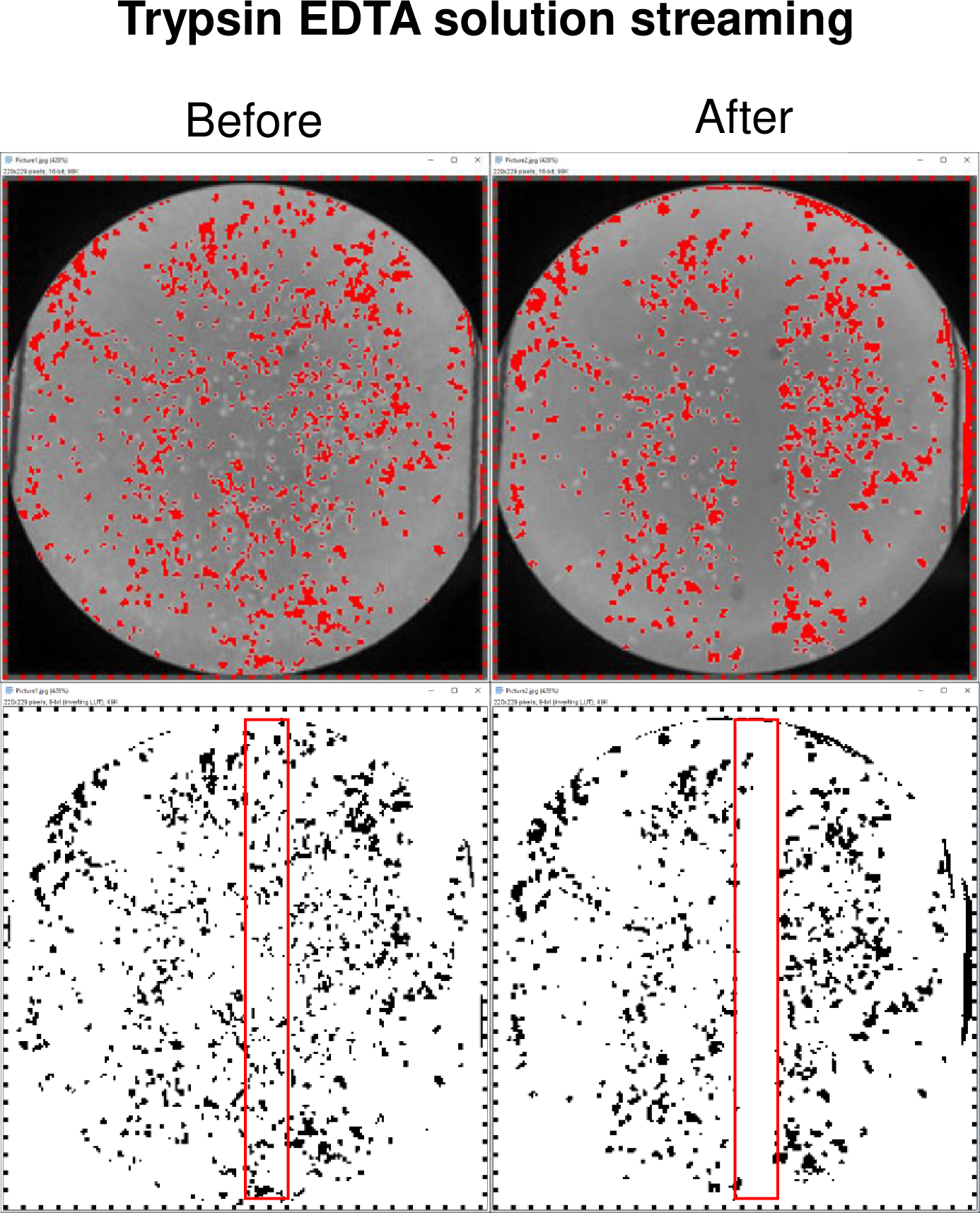

Supplement: S3 Fig — (TIF) [file pone.0279102.s003.tif]
