# Supplementary material for: Spatially selective cell treatment and collection for integrative drug testing using hydrodynamic flow focusing and shifting
Source: PLoS One. 2023 Jan 17;18(1):e0279102. doi: 10.1371/journal.pone.0279102 (PMC9844832; doi:10.1371/journal.pone.0279102)
Supplement: Attachment — Submitted filename: Response to Reviewers_PONE-D-22-11631.pdf [file pone.0279102.s004.pdf]

Point-by-point response to Editor's and Reviewers' comments

Spatially selective cell treatment and collection for integrative drug testing using hydrodynamic flow focusing and shifting

Xu Wang, Jingtian Zheng, Samantha C Emery, Maheshwar Adiraj Iyer, Adam Henry Szmelter, David T Eddington, and Steve Seung-Young Lee

Editor's and reviewers' comments:

We appreciate the Editor's and Reviewers' interests in our work and the opportunity to resubmit an improved manuscript.

Comment #1

Editor:

Both the Reviewers ask to expand the literature analysis. Similar microfluidic experiments are reported. It is very much desired that the reader can understand what are the technical advances in this paper and the differences with the published studies.

Reviewer 1:

First of all, similar microfluidic experiments were performed 10-20 years ago. The authors have not verified the technical advances in this paper, so it lacks novelty.

Reviewer 2:

I have only one suggestion, to better highlight the novelty of this work. I think the Authors should expand the literature survey at lines 51-57, providing more details about existing devices and approaches. In this way, the statement at lines 58-61 would be better motivated and supported.

-To clarify the technical novelty of our study, we have described more details of the previously reported hydrodynamic flow focusing devices and their applications in references 18-21. Based on the expanded literature analysis, we more clearly point out technical advances in our work. Please find the newly added sentences on lines 62-94 (highlighted in gray). In addition, to support the novelty of our microfluidic device design, we added simulation data to determine the optimal inlet geometry for effective control of the laminar flow position in S1 Fig, which was not presented in the initial submission. This new supporting data shows that the sample/side co-flow input channels without a junction is the optimal device inlet configuration for creating the narrowest width and precisely controlled path of the hydrodynamically focused and shifted sample streams, compared to other inlet geometries including cross-channel with an inverted T-shape junction and 45° tilted side channel with a down arrow-shaped junction. More detailed descriptions of the supporting data are on lines 96-101, 127-132 and 210-216 (highlighted in gray). Thus, our work has developed a novel microfluidic device for hydrodynamic flow focusing and shifting and demonstrated its potential for spatially selective drug delivery and integrative cell-based drug testing.

Comment #2

Editor:

Please, provide a characterization of the switching/stabilization of flow positions.

Reviewer 1:

The authors do not properly characterize the switching behavior of the flow position. In a laminar flow system such as the one in this paper, switching/stabilization of flow positions takes a certain amount of time, which is critically associated with the accuracy of cell processing. However, there is no description on this point.

To change the position of the sample stream, we first turned off the central sample input pump, then adjusted the flow rates of the left and right side fluids to preset the new position of the sample stream, and turned on the sample fluid input pump in the end. Therefore, we considered that the characterization of time-dependent concentration change of shifted sample stream right after turning on the input pump is important to demonstrate the capability of our microfluidic flow control for accurate cell-based drug testing. We added new simulation data in S2 Fig showing that it took 5 seconds for the shifted sample solution stream to reach the peak concentration right after turning on the input pump, while the mean width (264 ± 4 (SD) μm) of the sample stream was not noticeably affected during the time period. More detailed descriptions on the supporting data are on lines 217-227 (highlighted in gray). This supporting data provides a new characterization of our approach for hydrodynamic flow focusing and shifting.

Comment #3

Editor:

please, outline the advantages of the presented device over conventional cell culture systems.

Reviewer 1:

The system presented is not practical as a platform for evaluating the effects of drugs on cells. The advantages over conventional cell culture systems, including 96-well plates, are not described

We recognize that this comment is to clarify the advantage of an overall microfluidics approach for cell-based drug testing over conventional cell culture systems (like using a 96-well plate). To respond to the comment, we have added new sentences on lines 45-51 (highlighted in gray).

"Highly predictive preclinical drug testing is essential to increasing success rates in drug development [5]. Although incubation of cells with drug candidates in a multiwell format is the gold standard for high-throughput drug testing *in vitro*, static treatment conditions do not emulate the exposure of cells to drug molecules *in vivo*. In contrast, microfluidics systems can generate a controlled stream of drug solutions through microchannels where cells are located. This dynamic condition mimics an *in vivo* model with a tissue environment where cells interact and take up drug molecules being circulated in the bloodstream after administration [6, 7]."
