# Supplementary material for: Spatially selective cell treatment and collection for integrative drug testing using hydrodynamic flow focusing and shifting
Source: PLoS One. 2023 Jan 17;18(1):e0279102. doi: 10.1371/journal.pone.0279102 (PMC9844832; doi:10.1371/journal.pone.0279102)
Supplement: Attachment — Submitted filename: Response to Reviewers_PONE-D-22-11631R1.docx [file pone.0279102.s005.docx]

**Point-by-point response to Editor’s and Reviewer’s comments**

**Spatially selective cell treatment and collection for integrative drug testing using hydrodynamic flow focusing and shifting**

Xu Wang, Jingtian Zheng, Maheshwar Adiraj Iyer, Adam Henry Szmelter, David T Eddington, and Steve Seung-Young Lee

**Editor’s and reviewer’s comments:**

We appreciate the Editor’s and Reviewer’s interests in our work and the opportunity to resubmit an improved manuscript.

**Review Comments**

1. How did they connect between the syringe pumps and syringes? Please provide information about the tubing (or connection to the microfluidic device and syringes).

-The syringes were positioned on the pumps, as shown in the photography below. The tubing we used is Tygon® ND-100-80 (0.02 inch x 0.06 inch), one end was connected to the syringe needle and the other end was plugged into the hole of the PMDS layer of the device. We added a new sentence “**The syringes containing sample and buffer solutions were positioned on the pumps and the tubing (Tygon® ND-100-80 (0.02 inch x 0.06 inch)) was connected to the syringe needle and the other end was plugged into the hole of the PMDS layer of the device.**” on page 8, line 175 (highlighted in gray).

Syringe on a pump.

2. Did they treat the microchannel surface to adhere cells onto the microchannel? For the cell culture in the microfluidic device, how did they prevent drying out the cell culture medium? PDMS shows high gas permeability, and the microchannel may be dried out during the incubation. In addition, what is the confluency of the cells before each experiment?

-No, we did not treat the microchannel surface to adhere cells onto the microchannel, we find 4T1 cells adhere well to the glass substrate of the microchannel, and no further treatment was needed. Cell culture media in the device was changed every 12 hr, which can prevent drying out the cell culture medium (as described on page 7, line 157-163). No, microchannel did not dry out during the incubation, and confluency of the cells is more than 75% after 7 days of incubation, as shown in Fig 3 (as described on page 12, line 277)

3. On page 10, line 226, how did they measure the flow width?

-As mentioned in the manuscript, the concentration profiles of the sample fluid were simulated in the ‘COMSOL Multiphysics’ software (as described on page 8, line 166-167), and the mean flow width was determined by measuring the full width at half maximum (FWHM) of the simulation profile data (as described on page 10, line 219-220).

4. Why did they turn off the syringe pump for the sample stream? Generally, the flow rate could be changed without turning off the syringe pump.

-Turning off the syringe pump immediately stops of the sample solution flow, which prevents the treatment of unselected cells with drug or dye molecules during the positional shifting of sample stream. We added a new sentence “**This pump off-on method can prevent unselected cells from exposure to sample molecule during repositioning of sample solution stream.**” on page 10, line 223 (highlighted in gray).

5. On page 12, line 275, please elaborate the differences in growth rate compared with the typical cell culture method and other on-chip cell culture methods.

-We recognized we made overstatement ‘optimal environmental conditions….’ Yes, we didn’t optimize cell culture conditions in the HFSM device and also didn’t compare the cell growth rate with other on-chip culture methods because the growth of 4T1 cells in our device is enough to test our idea ‘spatially selective cell treatment and collection.’ So, we edited the sentence to ‘**This result demonstrates our device can provide proper environmental conditions for culturing and housing millions of adherent 4T1 tumor cells to examine spatially selective cell treatment and collection.”** on page 12, line 280-282 (highlighted in gray).

6. For the cell stain experiments, is there any effect of molecular (DiI) diffusion? In addition, when the flow rate was changed to shift the DiI stream, the DiD stream could stain undesirable cells onto the microchannel due to the shift of the flow stream. Please comment on it and how to prevent undesirable cell staining.

-Yes, there is an effect of molecular diffusion, but the effect is negligible because the diffusion rate is much slower than the flow rate of fluid in the device. To prevent undesirable cell staining and treatment during the positional shift of the flow stream, we turned off the syringe pump for the sample stream and turned on after adjusting the position as we described on page 10, line 223-225 (as the same answer for the question #4).

7. What is the cell recovery rate for the selective cell collection experiment?

-We could not determine the recovery rate for the selective cell collection due to the difficulty in accurate count of individual cells in the selected region in the device and cell suspension solution. But, here we briefly measured the change of area where 4T1 cells adhere in black dashed lines in Figure 4C, D before and after trypsin EDTA flow exposure using ImageJ software (in below). The segmented cell area between the red lines (in the 2^nd^ row images in blow) decreased by 79% (from 466 to 97 in pixel number) after trypsin EDTA flow exposure. It shows most cells in the region were detached by trypsin EDTA streaming. We added a new sentence and supporting figure “**Using ImageJ, we determined that the cell area of 79% between the black dashed lines in the Fig 4C, D was disappeared after trypsin EDTA flow treatment (S3 Fig.)**“ on page 14, line 321 (highlighted in gray).

8. For the selective cell collection and cytotoxic effect of doxorubicin, please provide the cell viability.

-With the goal of sequential assay of the cells treated with doxorubicin flow, we examined the proof of concept that our device permits selective cell collection using trypsin EDTA flow as shown in Fig 4E. So, we didn’t check the viability of non-drug treated cells. However, our microscopy imaging of collected cells after selective doxorubicin treatment displayed fluorescence signals of both doxorubicin and DAPI in cell nucleus (Fig 5B). DAPI is generally used to stain nucleus in dead cells due to its lower plasma membrane permeability. Therefore, the image data prove the death of the collected cells by cytotoxic effects of doxorubicin. This cytotoxic effect-driven cell death is also confirmed in Fig 6 showing that selective treated cells with doxorubicin were washed away in culture media change.

9. Please discuss the limitation of this device and method. Is there a possibility of cell type effects?

-Yes, there would be cell type effects for spatial cell collection in our device. 4T1 cells and other cancer cells have relatively higher culture dish binding and growth rates compared non-cancer cell lines such as neuron and vascular cells. For culturing a non-cancer cell line in our device, we might need to treat the glass substrate of the microchannel with cell adhesion enhancers such as poly-L-lysine or vacuum gas plasma. In this case, the treatment would negatively affect cell detachment by trypsin EDTA flow. So, it needs to have optimization to balance between cell attachment for culture and detachment for spatial collection. We addressed this limitation of current our device and experiment in the Conclusion as “**Although it requires further studies and optimization in cell culturing and spatially selective collection to expand the application of the device for other cell types..** “ on page 16, line 386.
